# Monocyte Transmodulation: The Next Novel Therapeutic Approach in Overcoming Ischemic Stroke?

**DOI:** 10.3389/fneur.2020.578003

**Published:** 2020-10-22

**Authors:** Joohyun Park, Ji Young Chang, Jong Youl Kim, Jong Eun Lee

**Affiliations:** ^1^Department of Anatomy, Yonsei University College of Medicine, Seoul, South Korea; ^2^Brain Korea 21 Plus Project for Medical Science, Yonsei University College of Medicine, Seoul, South Korea; ^3^Brain Research Institute, Yonsei University College of Medicine, Seoul, South Korea

**Keywords:** ischemic stroke, neuroinflammation, monocytes, monocyte conversion, macrophages

## Abstract

The immune response following neuroinflammation is a vital element of ischemic stroke pathophysiology. After the onset of ischemic stroke, a specialized vasculature system that effectively protects central nervous system tissues from the invasion of blood cells and other macromolecules is broken down within minutes, thereby triggering the inflammation cascade, including the infiltration of peripheral blood leukocytes. In this series of processes, blood-derived monocytes have a significant effect on the outcome of ischemic stroke through neuroinflammatory responses. As neuroinflammation is a necessary and pivotal component of the reparative process after ischemic stroke, understanding the role of infiltrating monocytes in the modulation of inflammatory responses may offer a great opportunity to explore new therapies for ischemic stroke. In this review, we discuss and highlight the function and involvement of monocytes in the brain after ischemic injury, as well as their impact on tissue damage and repair.

## Introduction

Stroke is the third leading cause of death globally, and ~80% of all strokes are ischemic strokes, which occur when brain cells die of reduced blood supply to various parts of the brain (1–3). Within minutes after the onset of stroke, immune processes are activated by ischemic cascades, from intravascular events triggered by the occlusion and moving of vessels to brain parenchymal inflammatory responses, leading to tissue damage and repair (4–10). The pathophysiological features of ischemic stroke, such as the production of reactive oxygen species (ROS) (11), the release of ATP and UTP by dying cells (12), and the loss of beneficial nitric oxide (NO) in states of oxidative stress (13), can trigger the activation of a neuroinflammatory response (14–17). In addition, cells that are dying of a lack of blood supply release cytokines, chemokines, and matrix metalloproteinases, thereby inducing the infiltration of blood cells, such as monocytes, lymphocytes, neutrophils, and natural killer cells, into the injured brain parenchyma (18–20). The initial immune response to neuroinflammation following ischemic stroke is characterized by early neutrophils swarming into the ischemic injured brain. Neutrophils' immune process has been extensively studied and is well-organized, with subsequent steps of cellular recruitment (21–25). However, little is known about the impact of monocyte recruitment to brain tissue affected by ischemic injury.

In mice, monocytes are categorized into two functional subpopulations, which can be differentiated by the expression of specific surface markers, such as lymphocyte antigen 6C (Ly6C), C-C chemokine receptor 2 (CCR2), and CX3C chemokine receptor (CX3CR1) (26). The first subset is characterized as short-lived and pro-inflammatory, with active recruitment to inflamed tissues by circulating through the blood; this subpopulation expresses Ly6C^+^CCR2^high^CX3CR1^low^ markers (27). The second subset is known for alternative and non-classical activation, and it is characterized by an anti-inflammatory phenotype and CX3CR1-dependent recruitment to local non-inflamed tissues; this subpopulation expresses Ly6C^−^CCR2^low^CX3CR1^high^ markers (28–30). Blood monocytes have plasticity and are embroiled in pro-inflammatory and anti-inflammatory responses in various diseases (28, 31–34). Blood monocytes are a heterogeneous population of circulating leukocytes. The classical pro-inflammatory monocytes (Ly6C^+^CCR2^high^CX3CR1^low^) are rapidly recruited to inflamed tissues and release high levels of inflammatory cytokines, such as tumor necrosis factor-α (TNF-α) (35), interleukin-1 β (IL-1β) (36), and IL-6 (37), when there is tissue damage. Further, these monocytes may turn into tissue macrophages (38). On the contrary, the alternative non-classical monocytes (Ly6C^−^CCR2^low^CX3CR1^high^) play a crucial role in reparative processes, producing anti-inflammatory cytokines, such as IL-4, IL-10 (39), and IL-13 (40), in inflammatory environments. Alternative monocytes can also turn into alternative macrophages (41–44). These two distinct subsets of monocytes are known to be polarized into M1 (pro-inflammatory) (45) or M2 (anti-inflammatory) (46) macrophages, thereby influencing the local milieu at different time points following tissue damage (47–49).

Given that the immune response comprises numerous cell populations that influence the neuroinflammatory process at different times, detailed analytical studies of infiltrating monocytes are needed. In this review, we summarize the precise roles of infiltrating blood monocytes on neuroinflammation following ischemic stroke, highlighting how the distinct subsets of monocytes work in beneficial and noxious ways. Efforts to understand the mechanisms of monocytes in neuroinflammation following ischemic stroke may not only help address the limitations of current stroke therapies, but also suggest novel treatment strategies for stroke through the modulation of immune cells.

## Development and Migration of Monocytes

**Figure 1:**
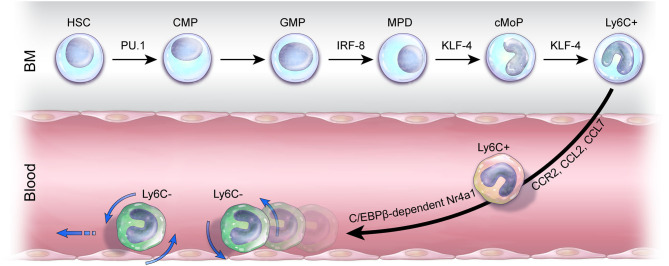
Monocyte development and mobilization. In the bone marrow (BM), pro-inflammatory Ly6C^+^ monocytes are derived from cMoPs that are differentiated from a series of hematopoietic stem cells (HSCs) by transcription factors PU.1, interferon regulatory factor 8 (IRF8), and Kruppel-like factor 4 (KLF4). Ly6C^+^ monocytes emigrate out of the BM in a C-C chemokine receptor 2 (CCR2)-dependent manner, and the ligands of CCR2, C-C motif ligand 2 (CCL2) and C-C motif ligand 7 (CCL7), stimulate Ly6C^+^ monocyte mobilization into the blood. The development of anti-inflammatory Ly6C^−^ monocytes from Ly6C^+^ monocytes depends on CCAAT enhancer–binding protein β (C/EBPβ)–dependent nuclear receptor subfamily 4 group A member 1 (NR4A1). Ly6C^−^ monocytes crawling on the endothelium in the periphery act in an immune surveillance capacity to maintain homeostasis.

Monocytes are distinct myeloid components of an organism's innate immune system, and they are heavily involved in organisms' neuropathological processes (50, 51). The development of classical Ly6C^+^ monocytes begins when they are derived from hematopoietic stem cells (HSCs) in the bone marrow (BM); depending on the macrophage colony-stimulating factor (M-CSF), the development of blood monocytes may vary (52, 53). Once Ly6C^+^ monocytes egress in the blood, they are considered incompletely differentiated cells, which have a high degree of developmental plasticity (54). These highly adjustable monocytes differentiate into phagocytic macrophages as they promptly drift toward inflamed tissues after sensing environmental stimuli following injury (55–57). In addition, in a steady state, circulating Ly6C^+^ pro-inflammatory monocytes are the precursors of the Ly6C^−^ monocytes patrolling the vasculature in the blood and BM (58). Furthermore, the differentiation and development of monocyte subpopulations are affected by numerous factors, including Kruppel-like factor 4 (KLF4) (59). Alder et al. investigated the consequences of KLF4 function loss for monocytes; the producibility of CD115^+^Gr^+^ monocytes, the Ly6C^+^ monocytes, was thoroughly reduced by the transplantation of KLF^−/−^ fetal liver cells into wild-type mice, which were subjected to lethal irradiation (59). CCR2 (60) and its agonists, monocyte chemoattractant protein 1 (MCP-1) (also known as CCL2) and MCP-3 (also known as CCL7), play a central role in the migration of Ly6C^+^ monocytes from BM to blood. The number of these classical monocytes was significantly decreased in the bloodstream, although it was significantly higher in CCR2 knockout mice compared to wild-type mice. Tsou et al. demonstrated that CCR2 ligands, MCP-1 and MCP-7, are some of the most necessary chemokines for proper control of monocyte levels (60). In addition, many studies regarding CCR2 signaling and chemokines related to Ly6C^+^ monocytes have been conducted for various diseases, suggesting that classical monocytes play a pivotal role in immune responses (61–64). By utilizing chromatin immunoprecipitation sequencing and gene expression profiling, researchers revealed that transcription factor KLF4 is a powerful indirect target gene of interferon regulatory factor (IRF8) (65). In addition, the differentiation of Ly6C^+^ monocytes was not observed in IRF8^−/−^ mice, suggesting that the IRF8-KLF4 axis is the key modulator in monocyte development (65). Transcription factor PU.1 (encoded by the *Spi1* gene), one of the erythroblast transformation–specific family transcription factors, is essential for transcriptional regulation in the development of the myeloid lineage (66). PU.1 is highly expressed in monocytes and macrophages (67). During monopoiesis, PU.1 promotes IRF8 expression in monocytic dendritic progenitors, whereas IRF8 directly binds to the promoter region of the *Klf4* gene in common monocyte progenitors (cMoPs), resulting in the differentiation of Ly6C^+^ monocytes (68). Hanna et al. investigated nuclear receptor subfamily 4 group A member 1 (Nr4a1, Nur77) (69), and by analyzing the monocyte population, they showed that Ly6C^−^ monocytes exhibit apoptosis in the BM of Nr4a1^−/−^ mice. Additionally, as shown through a series of adoptive transfer studies by Hanna et al., the conversion of Nr4a1^−/−^ Ly6C^+^ monocytes to Ly6C^−^ monocytes is aborted in the BM, blood, and tissues. This suggests that not only is Nr4a1 vital for Ly6C^−^ monocyte survival, but also differentiation of Ly6C^−^ monocytes from Ly6C^+^ monocytes is also crucial (69). Regarding transcription factor CCAAT enhancer–binding protein β (C/EBPβ), Mildner et al. confirmed that the two monocyte subsets were originally homogenous populations in the BM by transcriptome and epigenome profiling assays. However, heterogeneity of Ly6C^int^ monocytes in the blood, where the generation of Ly6C^−^ monocytes arising from Ly6C^+^ monocytes depends on C/EBPβ, is involved in regulating Ly6C^−^ monocyte survival factor Nr4a1 (70) (Figure 1).

## Functional Heterogeneity of Monocyte Subsets in Mice and Humans: The Vital Role of Microrna Level Detection

Monocytes, which consist of leukocytes circulating through the blood, are a heterogeneous population and a critical component of an organism's innate immune response against inflammation (14, 71). Although murine monocytes are often seen as dichotomous, monocytes in humans are divided into three distinct subsets, which are differentiated by the expression levels of CD14 (lipopolysaccharide receptor complex component) and CD16 (FCγRIII immunoglobulin receptor): (1) CD14^++^CD16^−^ (classical), (2) CD14^+^CD16^+^ (intermediate), and (3) CD14^+^CD16^++^ (non-classical) (72). When comparing murine and human monocytes, the classical and non-classical monocytes in humans are analogous to the Ly6C^+^ monocytes expressing CCR2^high^CX3CR1^low^ and Ly6C^−^CCR2^low^CX3CR1^high^ in mice (73). The third subset of monocytes in humans, the intermediate monocytes, is not comparable to any in mice and are thought to be in the middle stage of classical monocytes transitioning into non-classical monocytes (74, 75).

MicroRNAs (miRNAs), which are small non-coding RNAs, bind to specific messenger RNAs (mRNAs) and inhibit the translation or promote the degradation of mRNAs (76). They play a vital role in regulating monocyte development and function (77). From the extensive list of miRNAs, the miRNA investigated first and foremost for monocytes is miRNA-146a, which regulates the functional heterogeneity of monocyte subsets (78) and shows the largest difference in expression levels between Ly6C^+^ and Ly6C^−^ monocytes. Steady-state expression of miRNA146a is higher in Ly6C^−^ monocytes than in Ly6C^+^ monocytes in mice. In addition, miRNA-146a depletion induces abnormal myeloproliferation and myelodifferentiation of HSCs (79) and augments the pro-inflammatory responses of Ly6C^+^ monocytes without affecting the change of monocyte subset development (78). Through miRNome data analysis, differences in miRNA expression were quantified between classical and non-classical monocytes in humans. miRNA-17, miRNA-18a/b, miRNA-19b, miRNA-20b, and miRNA-106a were overexpressed in classical monocytes, whereas miRNA-132, miRNA-146a, and miRNA-342-3p were overexpressed in non-classical monocytes (80). Moreover, it was reported that different motility of monocyte subsets (81) is associated with the expression of miRNA-19a. Dang et al. showed that knockdown of miRNA-19a markedly blocked the movement of CD16^−^ monocytes, known as classical monocytes, suggesting that miRNA-19a plays a crucial role in promoting CD16^−^ monocytes motility (82). Further research also revealed that pre–miRNA-432 overexpression, which is one of the miRNA candidates selected by *in silico* analysis, induces apoptosis of CD16^−^ monocytes, and the expression of miRNA-432 was significantly different between CD16^−^ and CD16^+^ monocytes. This suggests that miRNA-432 regulates the apoptosis of CD16^−^ monocyte. In addition, Zawada et al. reported 38 miRNAs were differentially expressed in an intermediate subset of monocytes in humans, among which miRNA-150 was significantly downregulated in intermediate monocytes compared with both classical and non-classical monocyte subsets (83).

Recently, Chipont et al. reported that the number of Ly6C^−^ monocytes in the blood, BM, and spleen is significantly reduced in ApoE^−/−^ miRNA-21–deficient mice, resulting in the acceleration of the pro-inflammatory response in atherosclerosis. The study suggests that miRNA-21 inhibition in monocytes could be a plausible therapeutic approach in atherosclerosis (84). In addition, Selimoglu-Buet et al. revealed that miRNA-150 is a critical component in regulating the generation of the Ly6C^−^ monocyte subset in mice and humans (85). The authors demonstrated that unrepressed expression levels of the Tet methylcytosine dioxygenase 3 (*Tet3*) gene led to a decrease in the levels of Ly6C^−^ monocytes in miRNA-150–deficient mice, highlighting the clinical implications that diminished expression of miRNA-150 mostly found in the peripheral blood CD14^+^ monocytes of patients with chronic myelomonocytic leukemia. Given the studies discussed above, the detection of various miRNA levels in monocytes is needed to expand our knowledge of monocyte heterogeneity and develop new strategies to ameliorate monocyte-related diseases (Table 1).

**Table 1:** miRNAs-related monocyte heterogeneity.

**Monocyte functions**		**Pro-inflammatory**	**Anti-inflammatory**	**Anti-inflammatory**
**1. Heterogeneity of monocyte subsets related with miRNA expression in basal condition**				
Monocyte phenotype	Human	CD14^++^CD16^−^	CD14^+^CD16^+^	CD14^+^CD16^++^
	Mouse	Ly6C^+^CCR2^high^CX3CR1^low^	-	Ly6C^−^CCR2^low^CX3CR1^high^
Expression level of miRNAs	miRNA-17 miRNA-18a/b miRNA-19b miRNA-20b miRNA-106a miRNA-432 miRNA-150 miRNA-132 miRNA-146a miRNA-342-3p miRNA-19a miRNA-21	High High High High High Low High Low Low Low Low Low	ßLow Moderate Moderate Low Low Moderate Low Moderate Moderate Moderate Moderate Moderate	Low Low Low Low Low High High High High High High High
**2. Implications related to the role of miRNAs in monocyte subsets**				
miRNA-21 deletion	Increase of pro-inflammatory response			
miRNA-19a knockdown	Inhibition of CD16^−^ monocytes movement			
miRNA-146a deletion	Dysregulation of myeloproliferation and myelodifferentiation of HSCs			
	Dysregulation of pro-inflammatory response			
miRNA-150 deletion	Decrease of Ly6C^−^ monocyte by overexpression of *Tet3* gene			
miRNA-432 overexpression	Increase of CD16^−^ monocyte apoptosis			

## The Function of Microglia and Monocyte-Derived Macrophages: The Impact on Neuroinflammation Following Ischemic Stroke

### Microglia

Microglia, or resident tissue macrophages in the central nervous system (CNS), which account for 5 to 20% of the glial cell population (86), contribute to tissue homeostasis by interacting with neurons and surveilling the microenvironment of the brain under a steady state (87). Upon an ischemic cascade, microglia react to the danger signal and are rapidly activated within minutes after the onset of ischemic stroke. Following ischemic stroke, microglial proliferation peaks between 2 and 3 days after the onset of the injury and lasts for several weeks. Microglia are rapidly activated and can phagocytose cell debris (clear apoptotic cells) (88). Activated microglia have both beneficial and detrimental effects. From the beneficial point of view, microglial depletion caused by the administration of dual colony-stimulating factor 1 (CSF1)/c-kit inhibitor, PLX3397, may exacerbate post-ischemic neuroinflammation in the brain. In mice, microglial depletion can augment leukocyte infiltration, neuronal death, and inflammatory mediators, including IL-1α, inducible NO synthase, and TNF-α. This highlights that pathophysiological exacerbation depends not only on lymphocytes and monocytes, but also on inflammatory mediators and the action of astrocytes (89). In addition, the selective elimination of microglia results in significantly augmented infarct volume after ischemic insult, indicating the positive function of microglia (90). Moreover, minocycline treatment diminishes polymorphonuclear cells and inhibits microglial activation, which results in the attenuation of neuroinflammation in the ischemic injured brain (91). Furthermore, microglial depletion induced by liposome-encapsulated clodronate was shown to accelerate inflammation and brain injury by a surge of pro-inflammatory cytokines (e.g., IL-1β and TNF-α) and chemokines (e.g., MCP-1 and MIP-1α) in an acute postnatal (day 7) ischemic model (92). This, in turn, indicates that microglial contribution is a critical endogenous defense mechanism in ischemic stroke.

On the other hand, the deleterious role of activated microglia has also been reported. For example, microglia are more prone to releasing cytotoxic factors, such as ROS, NO, and TNF-α, in a severe ischemic environment compared to a mild ischemic environment (93). Furthermore, the preconditioning of isoflurane inhibits microglial activation, thereby reducing the infarct volume and attenuating neuronal apoptosis, supporting the detrimental role of microglia in neuroinflammation after ischemic stroke (94).

The copious amount of studies reporting controversial theories regarding the role of microglia in ischemic stroke reflects the importance of microglia and indicates the potential of exploring new therapeutic strategies related to microglia.

### Monocyte-Derived Macrophages

The process of monocytes homing in on inflamed tissue after ischemic stroke is a vital response for host defense. Before monocyte and leukocyte recruitment occurs at the injury site, cytokine and chemokine levels increase, contributing to ischemic stroke outcomes (95). After the onset of ischemic stroke, monocytes circulating in the blood infiltrate into the brain lesion through the disrupted blood–brain barrier (BBB) following the leukocyte cascade as a result of neuroinflammation (96–99). Monocyte infiltration is induced by several chemokines. Many researchers have focused their studies on MCP-1, which is also known as chemokine [C-C motif] ligand (CCL2) (100, 101). CCL2 is a potent chemokine-specific recruiter for monocytes, and its receptor, CCR2, is required for the egress of classical monocytes to the bloodstream from the BM. An increased level of circulating MCP-1 is associated with increasing the long-term risk of ischemic stroke (102), and the infiltration of CCR2^+^ monocytes is greatly diminished in mice with dysfunctional CX3CR1-CCR2 signaling, resulting in the attenuation of acute injury in a rodent model of childhood stroke (61). CCR2 is highly associated with the recruitment of pro-inflammatory monocytes to the ischemic brain (103). However, Yang et al. showed that remote ischemic limb conditioning (RLC) after ischemic stroke shifted peripheral blood monocytes to the CCR2^+^ pro-inflammatory monocytes subset, resulting in reduced acute brain infarct volume and brain swelling and improved functional recovery (104). The authors demonstrated that RLC-mediated protection against ischemic stroke injury is disrupted by the adoptive transfer of CCR2-deficient monocytes, highlighting the importance of the RLC-induced shift of monocytes into the CCR2^+^ monocyte subset in attenuating ischemic stroke outcomes. Recent studies have reported that long-term behavioral recovery was exacerbated by blocking monocyte recruitment following ischemic stroke in mice. To summarize, the number of monocytes recruited to the ischemic hemisphere peaks 3 days after stroke. Half of these infiltrating monocytes differentiate into pro-inflammatory macrophages (M1), whereas the other half exhibit an anti-inflammatory macrophage (M2) phenotype. However, treatment with CCR2 antibody, MC-21, drastically decreases the expression of anti-inflammatory genes, resulting in the abolishment of long-term behavioral recovery (105). In the same context, monocyte/macrophage deletion increases the M1/M2 polarization ratio of microglia, aggravating the ischemic stroke injury. These results indicate that infiltrating monocyte-derived macrophages may regulate microglial polarization by shifting excessive pro-inflammatory M1 polarization to the M2 restorative process of microglia (106).

It has been generalized that once resident microglia and infiltrating monocytes turn into macrophages in response to ischemic injury, it is hard to differentiate the origins of the macrophages, as they are morphologically similar (107). To overcome this matter, Tanaka et al. conducted a series of experiments in tracing monocytes/macrophages derived from BM. Chimeric mice expressed enhanced green fluorescent protein in a cerebral ischemic environment, revealing differences in the exact roles of resident microglia and monocyte-derived macrophages (108).

Meanwhile, in addition to the CCR2-CCL2 axis, another chemokine axis pertaining to monocytes in ischemic stroke has been reported. C-C motif ligand 5 (CCL5) inhibition induced by the addition of MKEY, an antagonist of CXCL4-CCL5 heterodimer formation, was shown to significantly reduce the infiltration of Ly6C^+^ monocytes, resulting in the attenuation of classical monocyte-mediated neuroinflammation in a model of experimental ischemic stroke (109). Moreover, repetitive hypoxic preconditioning prior to the onset of ischemic stroke, which induces upregulated CXCL12 levels, blocks leukocyte infiltration, thereby endogenously contributing to an anti-inflammatory phenotype (110). In contrast to Ly6C^+^ monocytes, Ly6C^−^ alternative monocytes have a different path of infiltration. CX3CR1 ligand fractalkine (CX3CL1), which is expressed in endothelial and neuronal cells (111), interacts with Ly6C^−^ monocytes that are patrolling the vasculature (112) and microglia that are surveilling the CNS environment. Both Ly6C^−^ monocytes and microglia highly express its receptor, CX3CR1 (113), under physiological conditions. At the onset of ischemic stroke, these interactions are disrupted, and as the level of CX3CL1 abnormally increases, Ly6C^−^ monocytes rapidly infiltrate the brain parenchyma (114), leading to microglial activation. The regulation of transforming growth factor β-activated kinase 1 (TAK1), which is involved in both innate and adaptive immune responses and has conflicting inflammatory effects depending on the cell type, plays a key role in monocyte infiltration into the injury site following ischemic stroke. TAK1 deletion leads to reduced monocyte infiltration, thereby improving outcomes after ischemic stroke (115). In addition, Werner et al. demonstrated that CXC motif chemokine receptor 4 (CXCR4) deficiency diminishes monocyte infiltration and dampens the levels of pattern recognition and defense response gene expression in monocyte-derived macrophages at the ischemic injury site, resulting in deteriorated ischemic stroke outcomes. These results indicate that CXCR4 distinguishing HSC-derived monocytes from microglia is a crucial factor in sustaining the beneficial role of monocytes through an innate immune system against neuroinflammation following ischemic stroke (116).

## Conversion of Monocytes After Ischemic Stroke

There has recently been a surge in interest regarding the origin of Ly6C^−^ macrophages. Early-infiltrating pro-inflammatory monocytes can differentiate into anti-inflammatory Ly6C^−^ wound macrophages (MONO to MØ) after ischemic stroke (117). In addition, CCR2^high^CX3CR1^low^ pro-inflammatory monocytes have been shown to turn into CCR2^low^CX3CR1^high^ anti-inflammatory macrophages (MONO to MØ) 3 days after ischemic stroke in CX3CR1^gfp/+−^CCR2^rfp/+^ functional transgenic mice using two-photon intravital imaging, indicating that monocytes play a notable role in neuroinflammation following ischemic stroke (118).

**Figure 2:**
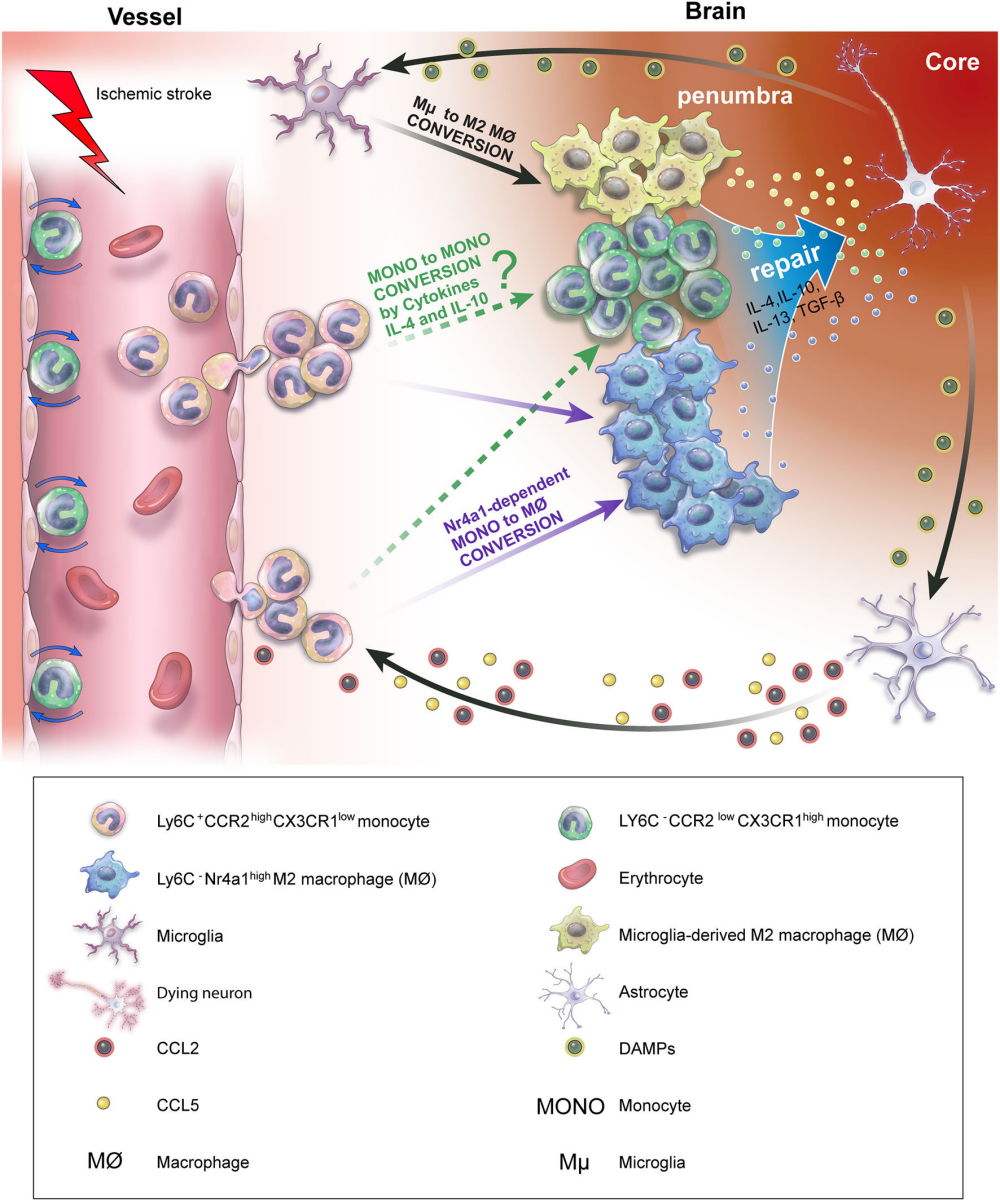
Ly6C^+^ pro-inflammatory monocyte conversion and contribution to ischemic stroke recovery *in vivo*. After the onset of ischemic stroke, DAMP released by dying neurons activates microglia and astrocytes. C-C motif ligand 2 (CCL2) and C-C motif ligand 5 (CCL5), which are secreted by activated microglia and astrocytes, induce the infiltration of Ly6C^+^ pro-inflammatory monocytes into the injury site. Infiltrating Ly6C^+^ monocytes convert into M2 macrophages depending on the presence of Nr4a1 (MONO to MØ). In addition, activated microglia are polarized into M2 macrophages, releasing anti-inflammatory cytokines, resulting in ischemic stroke recovery. However, whether the conversion of Ly6C^+^ pro-inflammatory monocytes to Ly6C^−^ anti-inflammatory monocytes (MONO to MONO) occurs in the brain, attenuating neuroinflammation following ischemic stroke, remains unclear.

Recently, the originally proposed dogma has been challenged in regard to the role of monocytes and monocytes differentiating into macrophages in the immune response. Jakubzick et al. revealed through parabiosis and bromodeoxyuridine pulse-chase analysis that monocytes can enter tissues and transmigrate to lymph nodes, maintaining their markers as monocytes, while not expressing macrophage or dendritic cell (DC) markers. In other words, classical monocytes are able to minimally differentiate into macrophages or DCs, surveying the non-lymphoid tissue and transporting antigens to lymph nodes under a steady state (119). However, this new concept of monocytes (MONO to MONO) has not been observed in the ischemic stroke environment (Figure 2). Future studies are warranted to examine whether the MONO-to-MONO conversion occurs after ischemic stroke.

## Monocyte Biomarkers in Clinical Studies of Ischemic Stroke

Abundant data have been accumulated regarding the overall pathologies of ischemic stroke. Based on these data, novel therapies, such as tissue plasminogen activator (tPA), are used to treat ischemic stroke. However, because several factors, such as sex, age, duration of ischemia, localization of the infarction, and possible comorbidities, must be evaluated for accurate ischemic stroke diagnosis, the treatments available today, such as thrombolytic agents, may only be effective for a narrow range of patients. Moreover, ischemic stroke is not a single neurodisease that leads to a local immune response; rather, it is a systemic inflammatory response that involves immune cells in the blood, making it more complicated to treat. To untangle and understand the complex systemic inflammatory response that follows ischemic stroke, monocytes are a popular research topic in the vast field of stroke. Today, the standard treatment for acute ischemic stroke (AIS) is thrombolytic therapy, which breaks down blood clots to restore blood flow in the brain (120). However, there are limitations to this treatment method, as it is only effective when the treatment is received within 3 to 4.5 h of symptom onset (121). As there is a narrow time frame for this therapeutic method, many patients are left with no alternative option. Therefore, much effort has been devoted to research new therapeutic methods to prevent the unfortunate outcomes of stroke (122, 123). To discover novel methods of ischemic stroke therapy, factors related to inflammation in the brain, such as C-reactive protein (CRP) (124), hepatocyte growth factor (125), insulin-like growth factor-1 (IGF-1), IGF-binding protein-3 (126), factor XIII (127), brain-derived neurotrophic factor (128), and d-dimer (129), have been investigated.

In addition, research on newly identified prognostic biomarkers related to monocytes in AIS has gained interest. Recently, Liu et al. reported that a higher monocyte-to-high-density lipoprotein ratio (MHR) is highly associated with the risk of poor functional outcomes within 3 months in patients with AIS, thereby suggesting MHR as a novel marker to evaluate ischemic stroke prognosis, widening the treatment window from mere hours to months (130). Wang et al. demonstrated the correlation between macrophage migration inhibitory factor (MIF) serum levels and ischemic stroke, stating that high MIF levels are directly related to moderate or severe ischemic stroke outcomes (131). Another emerging prognostic biomarker for AIS is the lymphocyte-to-monocyte ratio (LMR) (132). In a clinical study, Ren et al. assessed the stroke severity of 512 patients with AIS and 3 month outcomes using the National Institute of Health Stroke Scale (NIHSS) and conducted a series of experiments to evaluate relevant AIS markers, showing that a lower LMR is closely associated with severe AIS and poor outcomes. Similarly, a lower LMR is independently related to higher risk of hemorrhagic transformation (HT) in patients with AIS (133), suggesting that the LMR upon admission has good predictive value for AIS prognosis and may be used as a predictor for HT.

It has also been reported that T-cell immunoglobulin and mucin domain (TIM-4) expression levels in monocytes are remarkably elevated in patients with AIS after 2 and 5 days when compared to control patients. Importantly, TIM-4 expression levels in non-classical monocytes are highly correlated with NIHSS scores 2 days after stroke and are significantly increased in patients with AIS and poor outcomes, suggesting that the percentage of TIM-4 expression in non-classical monocytes could be a biomarker in predicting the clinical course and prognosis in AIS (134) (Table 2). In summary, numerous studies on biomarkers for the detection of ischemic stroke have been actively conducted in clinical practice; however, more work is needed to identify monocytes as a novel predictor of ischemic stroke. Therefore, research to discover biomarkers related to monocytes should be continued as it may lead to more promising stroke prognoses.

**Table 2:** Monocyte-related biomarkers of acute ischemic stroke (AIS) outcomes.

**Biomarkers**	**Biomarker levels indicating severe AIS**	*OR/**(*r*)	***P*-value**	**References**
MHR	High	2.58^*^	0.015	(130)
MIF	High	1.06^*^	<0.01	(131)
LMR	Low	0.523^*^	0.029	(133)
TIM-4	High	0.351^**^	0.048	(134)

*AIS, acute ischemic stroke; LMR, lymphocyte-to-monocyte ratio; MHR, monocyte-to-high-density lipoprotein ratio; MIF, macrophage migration inhibitory factor; TIM-4, T-cell immunoglobulin and mucin domain-4*.

*OR, *odds ratio*;

**r, *correlative coefficient*.

## Transcriptome Profiling of Monocyte/Macrophage in Ischemic Stroke: A Promising Gene-Targeting Therapy?

At present, there is no effective therapy against ischemic stroke and its sequelae. A variety of proteins have been studied (135) to confirm accurate ischemic stroke diagnosis and mitigate the neuroinflammation following ischemic stroke. Despite these efforts, successful therapeutics that are applicable to clinical studies regarding ischemic stroke have not been investigated.

Studies have recently been conducted in the field of stroke regarding the use of RNA in peripheral blood (136). These studies are supported by clinical applications in the diagnosis of diseases, such as myocardial infarction (137) and cancer (138). Brain transcriptomic analysis has been used in many experimental ischemic stroke studies to investigate specific changes in gene expression. These studies were mostly conducted in rodent models of ischemic stroke (139). As simultaneous blood and brain transcriptomics in patients with ischemic stroke is not feasible, Ramsay et al. conducted a series of experiments to analyze the whole gene expression pattern in both the blood and brain after ischemic stroke in a primate stroke model using *Rhesus macaque* (140).

Monocytes and macrophages infiltrate the ischemic injured brain and significantly impact ischemic stroke outcomes by regulating neuroinflammation. Hence, to investigate the exact role of monocytes and macrophages after ischemic stroke, current research trends have focused on transcriptome profiling (141).

Recently, Wang et al. performed genome-wide transcriptome profiling using RNA sequencing (RNA-seq) of monocytes and macrophages in the blood and brain samples of an ischemic stroke rodent model with distal middle cerebral artery occlusion (dMCAO) (142). Their findings indicated that ischemic stroke induced significant transcriptome changes in monocytes and macrophages in the post-stroke brain; however, only moderate changes were observed in the blood samples of the ischemic stroke group when compared with the control group. Further, macrophages in the post-ischemic stroke brain display unique transcriptome characteristics, robustly favoring neurovascular plasticity 5 days after ischemic stroke, suggesting that macrophages in the brain upregulate genes related with neovascularization, such as oncostatin M (*Osm*) (143), osteopontin (*Spp1*) (144), growth differentiation factor 15 (*GDF15*) (145), vascular endothelial growth factor (*VEGF*) (146), and fibroblast growth factor 1 (*FGF1*) (147). Surprisingly, lower angiogenesis and neurogenesis were verified in myeloid cell–specific peroxisome proliferator-activated receptor γ (PPARγ) knockout (mKO) mice than in wild-type mice 5 days after ischemic stroke. These results suggest that PPARγ, a master regulator of monocyte/macrophage genomic reprogramming, is a novel therapeutic target in determining reparative macrophage phenotypes and improving neurological function following ischemic stroke (142).

Another study by Zhang et al. revealed that PPARγ and signal transducer and activator of transcription 6 (STAT6) are potential upstream regulators of efferocytosis-related genes in macrophages, driving pro-efferocytic and anti-inflammatory phenotypes in the brain, thereby promoting ischemic stroke injury and recovery (148). To summarize, the blood-borne infiltrating monocyte/macrophage population, which is phenotypically expressed as CD11c-Ly6G^−^CD11b^+^CD45^high^ sorted by FACS in the brain, upregulates genes related with phagocytic activities, such as triggering receptor expressed on myeloid cells-1 (*TREM1*) (149) and extracellular signal–regulated kinase 5 (ERK5) (150), 5 days after dMCAO. In addition, genes involved in recruiting phagocytes, such as those encoding purinergic receptor 2 (*P2Y2*) and S1P receptor (*S1PR1*), were remarkably upregulated in brain monocytes/macrophages when compared with blood monocytes 5 days after dMCAO. Additionally, genes participating in the engulfment of apoptotic cells, such as *Abca1, Prkaca*, and *Rac1*, and representative cytoskeletal regulators (*Phldb2, Stmn1, Cit, Rac1, Cttn, Aurka*, and *Baiap2*) were significantly upregulated in brain macrophages compared with blood monocytes, suggesting that active engulfment signaling and cytoskeletal rearrangement in brain macrophages occur 5 days after dMCAO. Importantly, according to upstream regulator analysis, PPARγ and STAT6 may play pivotal roles in determining the pro-efferocytic transcriptome of macrophages in the brain 5 days after ischemic stroke (148). Regarding the transcriptome profiling, although the results could expand our knowledge of the exact role of monocytes and macrophages in the cerebral ischemic environment, the findings do not generalize all cases of ischemic stroke, as the results were only obtained from a single time point 5 days after ischemic stroke. Therefore, future studies may be warranted to confirm and examine the temporal profile of monocyte/macrophage genomic changes.

## Anti-Inflammatory Drugs Related to Monocytes and Macrophages for Treatment of Ischemic Stroke

Until recently, inflammatory monocytes recruited to the brain were believed to be representative mediators of neuroinflammatory injury and secondary neurotoxicity after ischemic stroke. Therefore, therapeutic intervention targeting CCR2 to prevent the recruitment of pro-inflammatory monocytes was considered to be beneficial against ischemic stroke, even though available data were controversial regarding monocytes roles after ischemic stroke (151, 152). These controversial aspects of monocytes were totally dependent on the severity and time window of the ischemic stroke, which suggested the importance of new therapeutics that could modulate the activity of monocytes in ischemic stroke. Current ischemic stroke treatments are focused on anti-inflammatory strategies that convert the complex characteristics of monocytes into anti-inflammatory proprieties following ischemic stroke.

Aspirin is a non-selective cyclooxygenase inhibitor that can prevent macrophage accumulation, resulting in cerebrovascular protection in the model of stroke-prone spontaneously hypertensive rats (SHRSP) (153). It has also been reported that aspirin delays the onset of ischemic stroke (153), providing clinical proofs of its protective effect against stroke in humans (154). Further, rosuvastatin, a widely used statin for lowering cholesterol and high-sensitivity CRP (155, 156), showed to attenuate the transcription of inflammatory biomarkers, such as MCP-1, IL-1β, TNF-α, TGF-β1, and p-selectin, while increasing the mRNA of endothelial NO synthase, overall supporting the anti-inflammatory processes in stroke-prone rats (157). Although it was reported that the use of statins is associated with augmented occurrences of hemorrhagic stroke in patients with a clinical history of cerebrovascular disease, they can be beneficial in preventing ischemic stroke (158). In the preclinical systematic review conducted by White et al., the PPARγ agonists thiazolidinediones, including rosiglitazone and pioglitazone, were found to suppress the cyclooxygenase-2 expression and promote the PPARγ DNA binding, resulting in the inhibition of inflammation after ischemic stroke (159). In addition, Nakamura et al. demonstrated that pioglitazone inhibits the infiltration of macrophages and suppresses the expression of inflammatory cytokines such as MCP-1 and TNF-α, thereby exerting a neuroprotective effect in SHRSP (160). Another anti-inflammatory drug targeting microglia and macrophages is minocycline, a known semisynthetic tetracycline antibiotic with antiapoptotic properties. It was demonstrated that minocycline inhibits activated microglia/macrophages that are involved in the demise of neurons and astrocytes, as well as endothelial cells in neurological diseases (161, 162). Evidence supporting the anti-inflammatory effect of minocycline showed that early treatment with this drug reduces the number of activated microglia/macrophage in the peri-infarct lesion of the ischemic injured brain, resulting in improved neurological outcomes after ischemic stroke in rats (163) (Table 3).

**Table 3:** Current ischemic stroke therapeutics targeting anti-inflammation.

**Therapeutics**	**Role(s)**	**Effect(s) on stroke**
Aspirin	- Non-selective cyclooxygenase inhibitor	- Decrease the accumulation of macrophages - Delays the onset of ischemic stroke
Rosuvastatin	- Lowers low-density lipoprotein cholesterol - Lowers high-sensitivity CRP levels	- Attenuate the expression of inflammatory biomarkers: MCP-1, IL-1β TNF-α, TGF-β1, p-selectin
Thiazolidinediones	- PPARγ agonists	- Suppress the cyclooxygenase-2 expression - Inhibit the infiltration of macrophages - Decrease the expression level of MCP-1 and TNF-α - Promote the PPARγ DNA binding
Minocycline	- Semisynthetic tetracycline antibiotic	- Reduce the microglia/macrophage activation - Improves the neurological outcome

From the strategies of ischemic stroke treatment currently available, the best therapeutic approach in the acute phase is thrombolysis with tPA; however, the therapeutic time window is extremely narrow (<4.5 h within stroke onset) (164). To expand the therapeutic window and, consequently, reach more patients with ischemic stroke, recent studies have focused on investigating immune cell–targeted therapies, such as anti-inflammatory drugs that are fundamentally non-invasive and effective approaches for ischemic stroke prevention (165). Despite substantial efforts to find an effective treatment for ischemic stroke through pharmacological modulation of immune cells, this still remains a challenge. As inflammatory mechanisms following ischemic stroke are complex, with multiple deleterious and protective effects, recent clinical trials of immunological therapies have failed (166–168). Importantly, most *in vitro* and *in vivo* studies regarding ischemic stroke were performed at the level of a single disease, not including comorbidities, such as hyperglycemia or diabetes. It was reported that most patients with stroke have comorbidities (169). Therefore, future studies should address ischemic stroke using models that mimic the disease, as well as its comorbidities, similar to patients, in order to develop more specific and accurate therapeutics.

As outlined above, recent emerging targets of monocytes transmodulation for ischemic stroke treatment have been focused on two factors, Nr4a1 and PPARγ (170, 171). As a vital factor for survival of non-classical monocytes, Nr4a1 plays an important role in the differentiation of Ly6C^+^ monocytes toward the anti-inflammatory phenotype of M2 macrophages, which has been proven in defective myocardial remodeling studies using Nr4a1^−/−^ mice (172). Furthermore, monocytes/macrophages polarization against neuroinflammation via pharmacological PPARγ activation in hyperglycemic and PPARγ (KO) mice resulted in brain repair with neovascularization in the infarct border zone after ischemic stroke. These findings suggest that Nr4a1 and PPARγ are strong modulators of Ly6C^+^ monocytes toward anti-inflammatory macrophage polarization, without affecting the infiltration of monocytes into the injured brain tissue.

**Figure 3:**
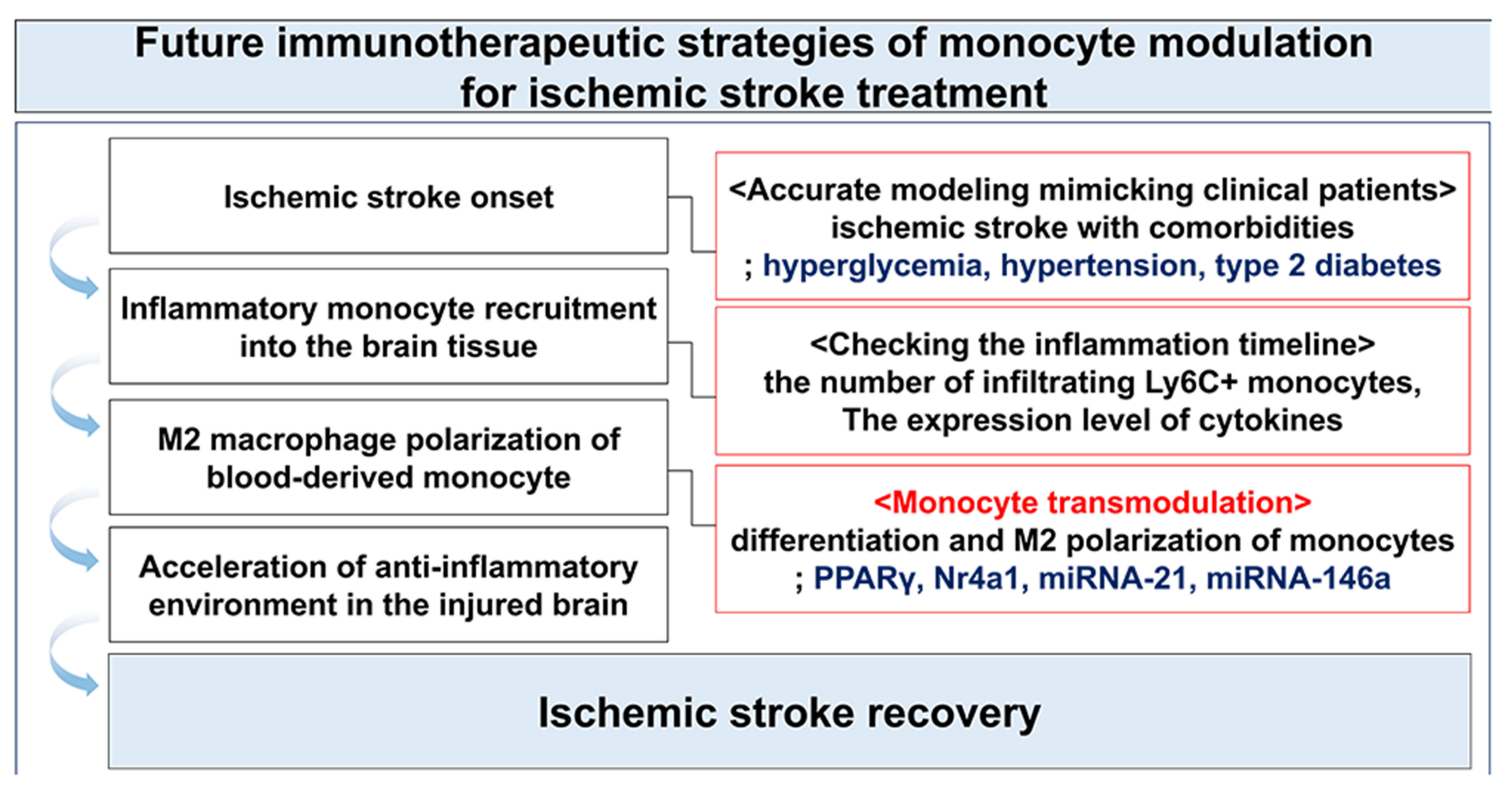
Immunotherapeutic strategy through the modulation of monocytes following ischemic stroke.

Past studies have intensively researched whether the infiltration of CCR2^+^ inflammatory monocyte or inhibition of CCR2 expression on monocytes affects post-ischemic stroke. Moreover, the role of inflammatory monocytes is known to vary in a time-dependent manner. Therefore, future therapeutic strategies may need to focus on increasing the polarization of infiltrating monocytes into macrophages with anti-inflammatory properties (M2) through modulation of factors such as Nr4a1, PPARγ, and miRNAs (e.g., miRNA-21, miRNA-146a) (Figure 3).

## Biotechnologies for Treatment of Ischemic Stroke: Current Advancements and Future Directions

**Figure 4:**
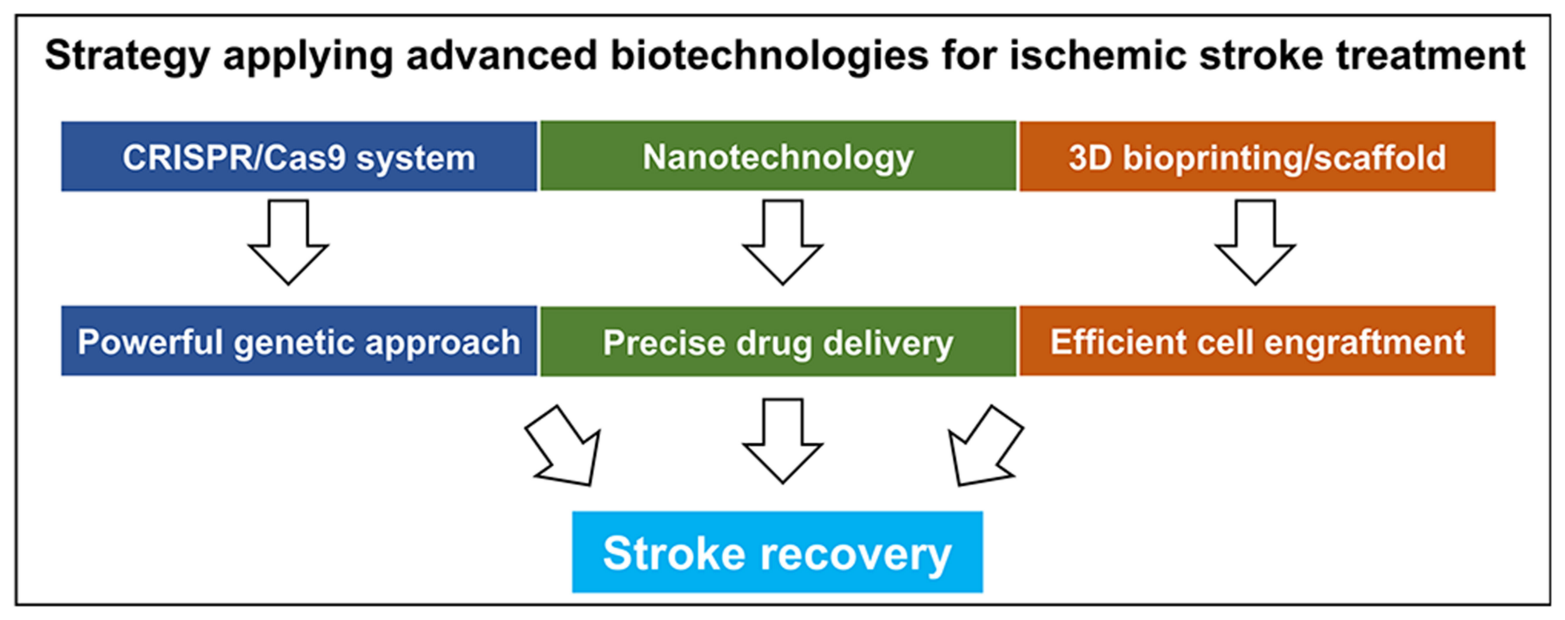
Current advanced biotechnologies for treatment of ischemic stroke.

Numerous studies in preclinical and clinical trials aim to discover functional recovery after ischemic stroke. From this point of view, immunoregulation by pharmacological drugs and neurogenesis stimulation, such as stem cell transplantation, have been considered as pivotal mechanisms in ischemic stroke treatment. However, despite these tremendous efforts to overcome ischemic stroke, no clear approaches for ischemic stroke therapy have been validated until now. With the recent advancements in science, various research regarding treatments of ischemic stroke using various cutting-edge technologies have been reported (Figure 4).

### CRISPR/Cas9 System

Clustered regularly interspaced short palindromic repeats (CRISPR)/Cas9 genome editing has emerged as a powerful tool that enables the correction of DNA mutation in both *in vitro* and *in vivo* models. CRISPR/Cas9 system is also known to be effective in creating gene KO and knock-in animal models for investigating the biological and pathophysiological mechanisms in various diseases (173). Recently, studies applying the advantages of CRISPR/Cas9 system have been reported in the field of stroke. It has been reported that the inhibition of semaphorin 4D (Sema4D), an axon guidance molecule, and its receptor, PlexinB1, which has the highest affinity to Sema4D, remarkably attenuate the inflammatory responses and BBB permeability and significantly reduce the infarct volume, resulting in the improvement of ischemic stroke outcome. The researchers in the study provided decisive evidence demonstrating the effect of Sema4D/PlexinB1 signaling using lentiviral transfection system targeting PlexinB1 disruption through CRISPR gene editing, which suggests that the regulation of Sema4D/PlexinB1 signaling is a novel therapeutic target for acute-phase treatment of ischemic stroke (174). Another study using CRISPR/Cas9 system in a genetic approach has been reported. Li et al. demonstrated that neuronal apoptosis was highly protected through Akt activation in ischemia-reperfusion (I/R) injury in Tollip-deficient rats, suggesting that Tollip is a novel modulator of I/R injury by promoting neuronal apoptosis and neuroinflammation (175). So far, despite the numerous advancements made through various genetic manipulations regarding the potential treatments of ischemic stroke, treatments targeting monocyte transmodulation using CRISPR/Cas9 system have not yet been reported. Therefore, future research to overcome this matter would be needed to continue as it may lead to more promising ischemic stroke treatment.

### Applied Nanotechnologies

As ischemic stroke occurs through different etiologies and pathophysiologies, the evolution of damage is complex and dynamic. Technologies based on structural and molecular brain imaging and effective drug delivery are necessary to apply precise and appropriate treatments at different phases. As a result, nanotechnologies, which involve nanomaterials, such as liposomes, nanocapsules, nanotubes, and micelles, have been developed for the treatment of stroke (176). Furthermore, to accurately assess a prognosis and overcome the difficulties limiting the present treatment of ischemic stroke, efforts to develop new biomarkers and investigate factors related to monocytes following ischemic stroke are ongoing in clinical studies. Recently, new assessments have been applied in clinical studies to improve the outcomes of ischemic stroke. Hou et al. reported the results of a new trial that showed that using monocytes and neutrophils, which were developed as carriers for cRGD [cyclo (Arg-Gly-Asp-d-Tyr-Lys)] liposome-mediated drug delivery, may alleviate ischemic stroke outcomes (177). Targeting monocyte that protects neuronal cell death through nanotechnology could be a novel therapeutic strategy for ischemic stroke. However, few optimal “nano” -related studies regarding immunomodulation of monocytes in ischemic stroke have been reported yet. Future studies are warranted to resolve the complexity of ischemic stroke in the context of nanotherapy targeting monocytes.

### Three-Dimensional Bioprinting

The main goal of ischemic stroke treatment is to promote the neurogenesis and angiogenesis, thereby improving the functional outcomes. However, few patients with ischemic stroke onset can be served timely with thrombolytic treatment, which mainly focuses on the rapid reperfusion, from the acute phase of occlusion. Therefore, to overcome the limitations, strategies ultimately promoting neurorecovery have been investigated (178). As a part of these therapeutic strategies, bioprinting, an emerging modern technology mixing cells related with tissue regeneration, such as stem cells, with biomaterials that are designed with high precision in order to obtain high compatibility with host tissues, has been developed (179, 180). Recently, it was reported that implanted exogenous human neural progenitor cells, which are electrically preconditioned on a conductive biopolymer scaffold prior to transplantation, cause the endogenous vasculature changes that enhance the gene expression involved in VEGF-A pathway, resulting in improvement of functional recovery after ischemic stroke (181). In addition, Brzezinski et al. demonstrated the efficacy of 3D printed novel holdfast devices. Briefly, left atrial appendage (LAA) occlusion is used as a method of preventing ischemic stroke in patients with atrial fibrillation. LAA exclusion device based on 3D printing using the selective laser sintering technology with polyamide powder was tested in this research because of its broad accessibility and low costs for production. The researchers tested for the reactions with local tissue and biocompatibility of the device when implanted in swine models. Results showed no clots on the atrium surface, and the foreign body reaction levels were similar to that of a polyester graft. This novel device that was evaluated in the research meets the biocompatibility parameters without any issues; that is, it is a suitable device for stroke prevention (182).

## Conclusion

During the past decade, significant progress has been achieved in the understanding and treatment of ischemic stroke. In particular, many studies have investigated the role of monocytes from various angles, including cellular and genetic approaches. The evidence discussed here suggests that the application of transcriptomics and epigenomics targeting monocyte/macrophage plays a crucial role in the regulation of neuroinflammation following ischemic stroke. The advancements in proteomics have remarkably contributed to development of novel biomarkers related to monocytes in evaluating the prognosis of ischemic stroke in clinical studies. The role of infiltrating monocytes in ischemic stroke is still controversial as it varies depending on timing and the degree of damage. This conflicting discussion supports vital evidence related to the importance of monocytes and the potential to convert into beneficial M2 macrophages by transmodulation. Several studies related to Ly6C^−^ alternative monocytes have also been reported in the field of ischemic stroke. Therefore, further research regarding alternative monocytes and the transmodulation of classical monocytes with the application of advanced biotechnologies is necessary, as it may open doors to new therapeutic strategies for stroke. Taken together, immunomodulation that aims to specifically control the functions of each monocyte subset is a curtail strategy for developing new therapies and may provide a promising future for the treatment of ischemic stroke.

## Author Contributions

JP and JC equally contributed to this study. This manuscript was written by JP and JC. JL and JK participated in the discussion and revision. The final manuscript was designed and edited by JL. All authors read and approved the final manuscript.

## Conflict of Interest

The authors declare that the research was conducted in the absence of any commercial or financial relationships that could be construed as a potential conflict of interest.

## Glossary

AIS, acute ischemic stroke; BBB, blood–brain barrier; BM, bone marrow; BrdU, bromodeoxyuridine; CCL2, chemokine [C-C motif] ligand 2; CCL7, chemokine [C-C motif] ligand 7; CCR2, C-C chemokine receptor 2; cMoP, common monocyte progenitors; CNS, central nervous system; CRP, C-reactive protein; CX3CL1, C-X3-C motif chemokine ligand 1; CX3CR1, C-X3-C motif chemokine receptor 1; CXCR4, C-X-C motif chemokine receptor 4; DCs, dendritic cell; dMCAO, distal middle cerebral artery occlusion; ERK5, extracellular signal–regulated kinase 5; FGF1, fibroblast growth factor 1; HSCs, hematopoietic stem cells; HT–hemorrhagic transformation; IGF-1, insulin-like growth factor-1; IL-10, interleukin 10; IL-13, interleukin 13; IL-1b, interleukin-1β; IL-4, interleukin-4; IL-6, interleukin-6; IRF8, interferon-regulatory factor 8; KLF4, kruppel-like factor 4; LMR, lymphocyte-to-monocyte ratio; Ly6C, lymphocyte antigen 6C; M-CSF, macrophage colony stimulating factor; MCP-1, monocyte chemoattractant protein-1; MHR, monocyte-to-high-density lipoprotein ratio; MIF, migration inhibitory factor; mKO, myeloid cell-specific PPARγ knockout; NIHSS, national institute of health stroke scale; NO, nitric oxide; NR4A1, nuclear receptor subfamily 4 group A member 1; Osm, oncostatin M; PPARγ, peroxisome proliferator-activated receptor γ; RLC, remote ischemic limb conditioning; ROS, reactive oxygen species; S1PR1, S1P receptor 1; STAT6, signal transducer and activator of transcription 6; TAK1, transforming growth factor-β-activated kinase 1; Tet3, Tet methylcytosine dioxygenase; TIM-4, T-cell immunoglobulin and mucin domain-4; TNF-α, tumor necrosis factor-α; tPA, tissue plasminogen activator; TREM1, triggering receptor expressed on myeloid cells 1; VEGF, vascular endothelial growth factor.
