# Corrigendum: Monocyte Transmodulation: The Next Novel Therapeutic Approach in Overcoming Ischemic Stroke?

**DOI:** 10.3389/fneur.2020.620560

**Published:** 2020-11-27

**Authors:** Joohyun Park, Ji Young Chang, Jong Youl Kim, Jong Eun Lee

**Affiliations:** ^1^Department of Anatomy, Yonsei University College of Medicine, Seoul, South Korea; ^2^Brain Korea 21 Plus Project for Medical Science, Yonsei University College of Medicine, Seoul, South Korea; ^3^Brain Research Institute, Yonsei University College of Medicine, Seoul, South Korea

**Keywords:** ischemic stroke, neuroinflammation, monocytes, monocyte conversion, macrophages

Shared first authorship between Joohyun Park and Ji Young Chang was mistakenly not mentioned in the authorship list. The corrected authorship, affiliation list and Author Contributions statement are indicated below.

Joohyun Park^1,2^^†^, Ji Young Chang^1,2^^†^, Jong Youl Kim^1^ and Jong Eun Lee^1,2,3^^*^

^1^Department of Anatomy, Yonsei University College of Medicine, Seoul, South Korea

^2^Brain Korea 21 Plus Project for Medical Science, Yonsei University College of Medicine, Seoul, South Korea

^3^Brain Research Institute, Yonsei University College of Medicine, Seoul, South Korea

^*^Correspondence:

Jong Eun Lee jelee@yuhs.ac

†These authors share first authorship

## Author Contributions

JP and JC equally contributed to this study. This manuscript was written by JP and JC. JL and JK participated in the discussion and revision. The final manuscript was designed and edited by JL. All authors read and approved the final manuscript.

The authors apologize for this error and state that this does not change the scientific conclusions of the article in any way. The original article has been updated.

